# Use of mammals in a semi-arid region of Brazil: an approach to the use value and data analysis for conservation

**DOI:** 10.1186/s13002-019-0313-4

**Published:** 2019-07-09

**Authors:** Suellen da Silva Santos, Reinaldo Farias Paiva de Lucena, Hyago Keslley de Lucena Soares, Vanessa Moura dos Santos Soares, Natalice Santos Sales, Lívia Emanuelle Tavares Mendonça

**Affiliations:** 1 Universidade Federal da Paraíba, João Pessoa, Brazil; 2 UEPB, Campina Grande, Brazil; 3 PRODEMA, João Pessoa, Brazil; 4 Campus I, Center of Exact and Natural Sciences, Department of Systematics and Ecology, Laboratory of Ethnobiology and Environmental Sciences, Federal University of Paraíba, João Pessoa, Brazil; 5 UFRJ, Rio de Janeiro, Brazil; 6 Biology Department, State University of Paraíba, João Pessoa, Brazil

**Keywords:** Animal use, Ethnomastozoology, Use value, Current use value

## Abstract

**Background:**

This study aimed to survey the knowledge and use of mammals by the residents of the rural community of Capivara in the municipality of Solânea (Paraíba State, Northeast Brazil) and to propose a new method of using the use value as a tool for data analysis in ethnozoological surveys.

**Methods:**

The uses attributed to mammals were recorded through semi-structured interviews conducted with the breadwinners (men and women) living in the community. The species were identified through guided tours, by descriptions made by the interviewees, and using specimens donated by them, as well as by comparison with the pertinent scientific literature (morphological and ecological). Through the use value differentiated analysis, it was possible to distinguish the current use value of the species (effective use) from their potential use value (knowledge, but no effective use) to determine their real importance related to the uses cited by the studied group.

**Results:**

Nineteen species were cited; however, only 17 of them were identified and then distributed in 13 families. The other species were identified at the genus level *Leopardus* sp. and order Rodentia. The species were classified into 6 categories of use: food, captive breeding, zootherapeutic, artisanal, magic/religious, and veterinary purposes.

**Conclusions:**

This article discusses possible conservation solutions, given the irregular exploitation of some species, warning about the biodiversity, and traditional knowledge conservation.

## Background

The biological diversity of mammals in Brazil, described by Paglia et al. [1], comprises 701 species distributed in 243 genera, 50 families, and 12 orders. In the semi-arid region, Albuquerque et al. [2] recorded 156 species occurring in the Caatinga. This biome has gotten the attention of researchers focusing on mammal studies, with some specific surveys aimed at obtaining data on the richness, ecology, ethology, physiology, distribution, and taxonomy of species (e.g., [3–9]).

Regarding the traditional use of mammals, a growing number of studies have been carried out in Brazil (e.g., [10–13]). In the Caatinga biome, some species of mammals have been used for food, pets, medicinal, magic/religious, artisanal, veterinary (folk medicine used in animals), and control (slaughter of wild animals that feed on domestic species) purposes (e.g., [14–19]). Animals that have utility value for the population are mostly killed in hunting activities, which, according to Chiarello et al. [20], represent one of the greatest threats to wild mammals. Consequently, this activity has caused a significant decline in several species’ populations throughout the world [21–25], intensifying the process of fauna extinction.

In order to understand the dynamics of use of the local fauna by the populations, data collection techniques have been diffused and currently used by different researchers. Among these techniques, the use value index (UV) stands out as a quantitative method, proposed by Phillips and Gentry [26, 27], in ethnobotanical studies, and adapted by Rossato et al. [28]. This method makes it possible to test hypotheses in ethnobiological research, measuring the importance of each species at the local and regional level, according to the interviewees.

The UV is used in several ethnobotanical studies (e.g., [29–32]) and, currently, also in ethnozoological research (e.g., [33–36]) to analyze the relative importance of a given species. However, because it does not distinguish current use (effective use) from potential use (knowledge, but no effective use), this index has limitations, evidenced in some ethnobotanical studies [29–31, 37]. According to La Torre-Cuadros and Islebe [29], a plant, for example, can receive many citations of use without being currently used in the analyzed population. Stagegaard et al. [38] also show that the UV is fragile since it assesses a high number of potential uses, considering that the species under this perspective have no effective use.

Therefore, Lucena et al. [39] proposed, in their studies in the Brazilian semi-arid region, a change in this index and developed the current use value (UV_current_), based on the uses that people reported as effective (currently executed by them), the potential use value (UV_potential_), based on the uses that people stated to know, but are not currently executed by them, and the general use value (UV_general_), commonly used in the literature and does not distinguish between effective use and knowledge.

Currently, many studies involving local populations [10, 40–43] have shown that mammals are the main sources of protein in the tropical regions of the world, and this is the main factor that promotes the capture and slaughter of these animals. Thus, the preference of human populations for large- and medium-sized species was also analyzed based on the Optimal Foraging Theory, which is a model of evolutionary ecology that has been used in the analysis of subsistence of human populations in several studies [44, 45]. This theory predicts that the animal will try to maximize the amount of resource obtained (benefit) per unit of time spent on foraging (cost) [46, 47].

However, some studies [40, 48–50] have shown that when there is a lack of preferred species, hunters have to capture a higher number of prey of less appreciated species, as well as to increase the invested time and cover a larger area to capture the animals. It is noteworthy that several cultural factors (taboos involving the choice of species and/or hunting areas, and forms of use) are involved in the selection, capture, and use of species by local populations and have considerable impacts on the populations of the species used [41–43, 48, 51]. In this sense, the understanding of these factors is essential to subsidize actions aimed at the management and conservation of the animals used [41, 43, 52].

Thereby, this study recorded and evaluated how wild mammals are used by residents of a rural community in a semi-arid region of northeastern Brazil. It was assumed that the biomass is a determinant factor in the choice and use of wild mammals by traditional/local populations. Thus, the following hypotheses were tested: (1) The higher the biomass of a species, the higher its use value and (2) the calculation of the differentiated use value modifies the list of the locally most important species.

## Materials and methods

### Study area

**Fig. 1:**
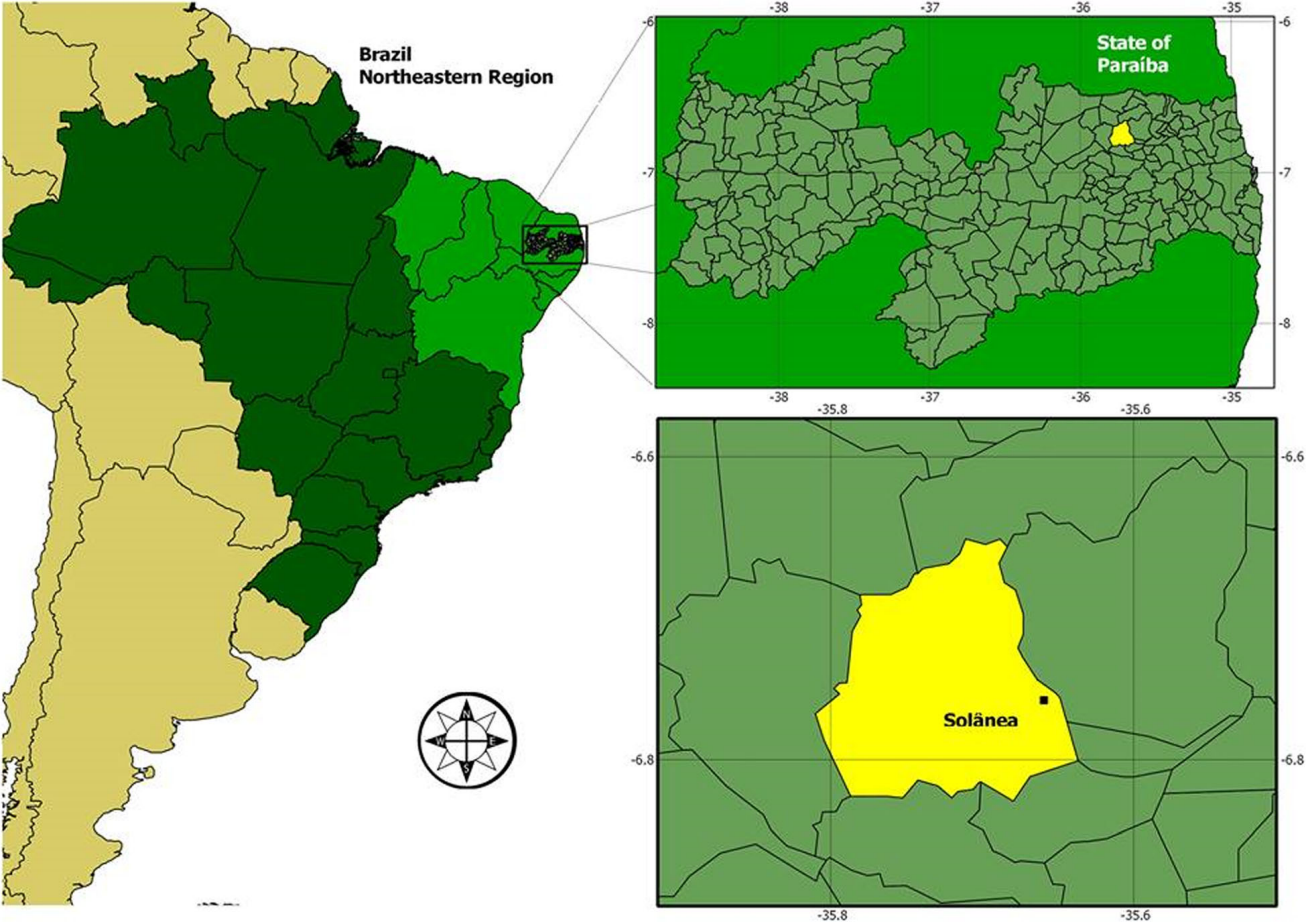
Map of the municipality of Solânea, state of Paraíba, northeastern Brazil. (Map: Natan Medeiros Guerra, 2014)

This study was carried out in the rural community of Capivara, municipality of Solânea (latitude 06° 45′ 18″ S longitude 35° 32′ 24″ W), inserted in the geoenvironmental unit of the “Borborema” Plateau, located in the “Agreste” Mesoregion and in the Solânea Microregion, in the semi-arid region of Paraíba State, Northeast Brazil, at 99.3 km from João Pessoa, the state capital, and can be accessed via Highways BR 230/BR 041/PB 105 [53] (Fig. 1). This municipality has a population of 26,925 inhabitants distributed in an area of 232,096 km^2^; 7361 of them live in rural areas (3699 women and 3662 men), and about 17,273 are literate [54].

### Data collection

This research was carried out in 2012, through several visits to the community for data collection and other observations. All the residences of the chosen sample were visited, but not all the family breadwinners were found, even after repeated visits to their residences. Therefore, 108 informants were interviewed (52 men and 56 women).

Information on the use of mammals in the region was obtained through semi-structured questionnaires, complemented by free interviews and informal conversations [55]. The participants’ socioeconomic data were also recorded during the interviews (Table 1).

**Table 1:** Socioeconomic profile of the respondents

Gender	
Men	52
Women	56
Age group	
19–30	16
31–50	42
51–70	34
> 70	1
Not informed	15
Residency time	
> 60 years	2
31–60 years	14
15–30 years	51
< 15 years	35
Not informed	6
Schooling level	
Semi-illiterate	16
Illiterate	34
Incomplete primary education	35
Complete primary education	4
Incomplete secondary education	2
Complete secondary education	2
Not informed	15

Before each interview, the participants were explained about the aims of the study and then were asked to sign the informed consent form, which is required by the National Health Council through the Ethics Research Committee (Resolution 466/12) and approved by the State University of Paraíba (protocol No. 45051115.5.0000.5187). The interviews dealt both with the population’s socioeconomic status and information on the mammals in the region such as the purposes for which they are used, their respective parts used, animal capture techniques, and morphological and ecological descriptions of the species.

In an attempt to obtain reliable answers from the interviewees, a friendly dialogue was held at the first contact with the participants, dealing with topics such as the pleasure of contact with nature, the taste of wild animal meat, and the experience transmitted through generations. As the informal conversation was held in a relaxed manner, the questions from the semi-structured questionnaire were asked. In addition, there was more than one contact with the residents to strengthen the bonds of trust between researcher and informant.

Guided tours were conducted during the informants’ daily activities and non-participant observation was carried out [56] for a more precise recording of the animal capture techniques, the preference for species, and other relevant information.

To mitigate the possible effects of the non-recording or non-veracity of information, the synchronous confirmation of the information was made, checking the data obtained from one informant with those obtained from others.

The species vernacular names were recorded exactly as mentioned by the interviewees, and the species were identified as follows: (1) analysis of the specimens or their parts donated by the informants, (2) analysis of animals pictures taken during the interviews and during the monitoring of hunting activities, (3) based on vernacular names, with the help from taxonomists who knew the fauna of the study (researchers from the Federal University of Paraíba - Campus I - Mastozoology Laboratory), and (4) based on ethnozoological studies previously performed in the study mesoregion [12, 13, 16, 57].

### Data analysis

The UV was applied to quantify the local importance of a species according to the interviewees [28]. In the present study, this index was calculated based on Lucena et al. [39], considering three types of data: uses that people cited as effective (known and currently applied by them) (UV_current_), uses that people were aware of but do not themselves use (UV_potential_), and the general use value (UV_general_) referred to uses that were commonly reported in the literature but with no distinction between use and knowledge [28]. This distinction was made during interviews by asking the interviewees to indicate uses that were effective (current) or not (potential). These values were calculated using the Microsoft Excel software (2012).

$$ {\mathrm{UV}}_{\mathrm{current}}={\mathrm{Ui}}_{\mathrm{current}/\mathrm{n}} $$

where UV_current_ = current use value of the species, Ui = number of citations of current use of the species mentioned by each informant, *n* = total number of informants.

$$ {\mathrm{Uv}}_{\mathrm{potential}}={\mathrm{Ui}}_{\mathrm{potential}/\mathrm{n}} $$

where UV_potential_ = potential use value of the species, Ui = number of citations of potential use of the species mentioned by each informant, *n* = total number of informants.

$$ {\mathrm{Uv}}_{\mathrm{general}}={\mathrm{Ui}}_{\mathrm{general}/\mathrm{n}} $$

where UV_general_ = general use value of the species, Ui = number of citations of general use of the species mentioned by each informant, *n* = total number of informants.

The counting of the animal species citations was made considering information such as preparation methods, therapeutic indications, and artisanal purposes, and for each different kind of use of the same species, an additional citation was counted.

For example, regarding food category, the citations for the use of cooked or roast meat were counted as distinct uses, i.e., cooked meat was considered one kind of use and roast meat another (a citation for each one). As for the medicinal animals, the citations were related to the specimen’s parts used and to the diseases treated by their uses. Therefore, when the melted animal fat is used to treat uterine problems, bone inflammation, throat inflammation, and toothache, four different citations of use are counted. If from this same specimen, another biological part is used for other therapeutic indications, more uses are attributed to it.

With regard to the artisanal use, the citations were counted according to the specimen’s biological part used (e.g., leather, carapace) and to the purposes attributed to it, such as for tambourines, zabumba, saddle, chair seat, sandal sole, seats for motorcycles, and belts, which are different items representing a citation each. This logistics is used as a model for the other categories of use, except for breeding and control since there is no diversity in their description.

The one-way ANOVA was used to identify significant differences between the results from the three types of use value calculated for each species, and the Tukey test was applied to identify which means were statistically different between each other (*p* < 0.05 and *p* < 0.01). The data analysis was performed in the Past 2.17c. Furthermore, the similarity analysis (cluster) was performed to analyze the similarity between these UVs, using the PRIMER 6.0 software.

To test if the biomass of the species predicted the use value attributed to them, a simple linear regression was made, adopting the UV_current_ as a dependent variable and the biomass of each species as an independent variable, using the BioEstat 5.0 software.

Biomass was calculated by multiplying the number of captured animals by the medium body mass of the species, found in the literature on mammals [58–60].

## Results

The interviewed residents cited 19 specimens; 17 of them were identified at the species level and distributed in 13 families, the others were identified at the genus level *Leopardus* sp. and order Rodentia.

Several kinds of use were attributed to the species, which were distributed in 6 categories: food (19 species), breeding (12 species), artisanal (11 species), zootherapeutic (9 species), magic/religious (1 species), and veterinary purposes (1 species) (Table 2).

**Table 2:** Ordering of the ten most important local species, based on the use value (UV), in the rural community of Capivara in the municipality of Solânea (Paraíba State, northeastern Brazil)

Scientific name	Vernacular name	UV_general_	UV_current_	UV_potential_
Primates				
Cebidae				
*Callithrix jacchus* (Linnaeus, 1758)	Common marmoset	–	–	10°
Carnivora				
Canidae				
*Cerdocyon thous* (Linnaeus, 1766)	Fox	4°	9°	3°
Felidae				
*Leopardus sp.*	‘Polka dot cat’ / Wild cat’	3°	8°	2°
*Puma yagouaroundi* (É. Geoffroy Saint-Hilaire, 1803)	Jaguarundi (Red Cat / Black Cat / Blue Cat)	2°	9°	1°
Mephitidae				
*Conepatus semistriatus* (Boddaert, 1785)	Skunk	7°	4°	5°
Cingulata				
Dasypodidae				
*Euphractus sexcinctus* (Linnaeus, 1758)	Yellow armadillo	7°	5°	4°
Rodentia				
Caviidae				
*Galea spixii* (Wagler, 1831)	Prea	1°	1°	5°
*Kerodon rupestris* (Wied-Neuwied, 1820)	Rock cavy	6°	3°	6°
Echimyidae				
*Thrichomys laurentius* (Thomas, 1904)	Punaré rat	5°	2°	9°
Lagomorpha				
Leporidae				
*Sylvilagus brasiliensis* (Linnaeus, 1758)	Forest rabbit	10°	10°	–
Pilosa				
Myrmecophagidae				
*Tamandua tetradactyla* (Linnaeus, 1758)	Collared anteater	9°	7°	8°
Didelphimorpha				
Didelphidae				
*Didelphis albiventris* Lund, 1840	Brazilian opossum	8°	6°	7°

It was noticed that the informants (men and women) are involved in pre- and post-hunting activities and that the men are responsible for obtaining the resources by hunting, making products used in popular veterinary and for magic/religious purposes (popular belief). The women are in charge of making food and zootherapeutic products. However, both men and women are involved in activities relating to the breeding of wild species.

**Fig. 2:**
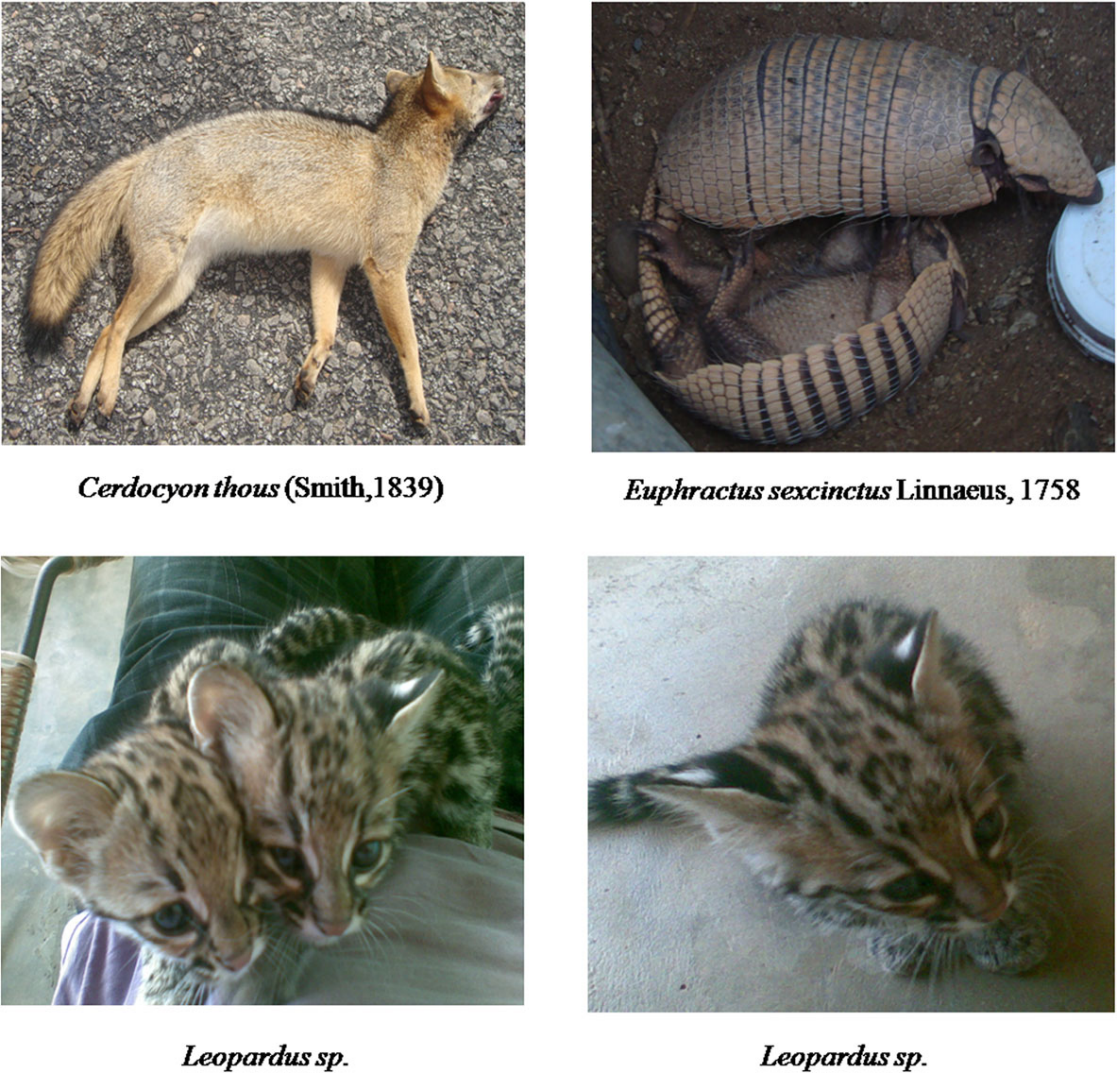
Photographic records of species cited by the residents of the rural community of Capivara in the municipality of Solânea (Paraíba State, northeastern Brazil)

*Galea spixii* (Wagler, 1831) (226 citations), *Puma yagouaroundi* (É. Geoffroy Saint-Hilaire, 1803) (190 citations) and *Leopardus sp.* (182 citations) had the highest number of citations. Regarding the utility value, *G. spixii* had the highest general use value (UV_general_ = 2.09), followed by the carnivore species *P. yagouaroundi* (UV_general_ = 1.76) and *Leopardus sp.* (UV_general_ = 1.68). The rodents *G. spixii (*UV_current_ = 1.49), *Thrichomys apereoides* (Lund, 1941) (UV_current_ = 1.12), and *Kerodon rupestris* (Wied-Neuwied, 1820) (UV_current_ = 0.75) had the highest current use (effective use) values. This high UV_current_ indicates that possibly these species have been exploited locally. *P. yagouaroundi* (UV_potential_ = 1.50), *Leopardus sp*. (UV_potential_ = 1.36), and *Cerdocyon thous* (Linnaeus, 1766) (UV_potential_ = 1.36) were the most prominent species regarding the potential use value (Tables 2 and 3) (Fig. 2).

**Table 3:** Ordering of species of local importance, based on their use value (current, potential and general), their respective use categories (*Fo* food, *Cb* captive breeding, *Mag/Rel* magic/religious, *Zoot* zootherapeutic, *Art* artisanal, *Vet* veterinary), zootherapeutic uses (used parts and treated diseases), and number of citations attributed to greater perceived abundance described by the residents of the rural community of Capivara, in the municipality of Solânea (Paraíba State, northeastern Brazil)

Scientific name	Vernacular name	Purpose	UV_general_	UV_potential_	UV_current_	Used part	Treated disease	Perceived abundance
Primates								
Cebidae								
*Callithrix jacchus* (Linnaeus, 1758)	Common marmoset	Fo; Cb	0.22	0.18	0.04	–	–	20
Carnivora								
Canidae								
*Cerdocyon thous* (Linnaeus, 1766)	Fox	Fo; Cb; Mag/Rel; Zoot; Art; Vet	1.42	1.16	0.26	Fat (2, 5) Leather (1) Liver (7)	Uterine problems Hemorrhoids Bone inflammation Gastritis Ulcer Throat inflammation Toothache Hemorrhage Rheumatism General pains Asthma Infection The Flu Low uterus Infertility Pregnancy problems Cancer Worms	53
Felidae								
*Leopardus pardalis* (Linnaeus, 1758)	Ocelot	Fo; Art	0.04	0.04	0	–	–	–
*Leopardus spp.*	“Polka dot cat”/“Wild cat”	Fo; Cb; Zoot; Art	1.68	1.36	0.32	Leather (1)	Inflammation	20
*Puma concolor* (Linnaeus, 1771)	Puma	Fo; Art	0.04	0.04	0	–	–	–
*Puma yagouaroundi* (É. Geoffroy Saint-Hilaire, 1803)	Jaguarundi (Red Cat/Black Cat/Blue Cat)	Fo; Cb; Zoot; Art	1.76	1.50	0.26	Leather (1)	Inflammation	26
Mephitidae								
*Conepatus semistriatus* (Boddaert, 1785)	Skunk	Fo; Zoot	1.10	0.60	0.50	Bones (7, 4) Meat (8) Liver (6)	Back pain Rheumatism Diabetes	25
Mustelidae								
*Galitictis cuja* (Molina, 1782)	Lesser grison	Fo; Cb; Art	0.10	0.08	0.03	–	–	13
Procyonidae								
*Procyon cancrivorous* Storr,1780	Crab-eating raccoon	Fo; Art	0.13	0.13	0	–	–	21
Rodentia	Rodents—Rat / ‘Fava bean rat’	Fo	0.03	0.02	0.01	–	–	10
Caviidae								
*Kerodon rupestris* (Wied-Neuwied, 1820)	Rock cavy	Fo; Cb; Zoot; Art	1.19	0.44	0.75	Fat (2) Meat (3) Intestine (4) Skin (4) Foot leather (4)	For eye cleansing Weakness Shortness of breath	12
*Galea spixii* (Wagler, 1831)	Prea	Fo; Cb; Zoot	2.09	0.60	1.49	Bones (6) Fat (5) Head (3)	Mumps Rheumatism For teeth strengthening	39
Cuniculidae								
*Cuniculus paca* (Linnaeus, 1766)	Paca	Fo	0.02	0.02	0	–	–	–
Echimyidae								
*Thrichomys laurentius* (Thomas, 1904)	Punaré rat	Fo; Cb	1.38	0.26	1.12	–	–	25
Lagomorpha								
Leporidae								
*Sylvilagus brasiliensis* (Linnaeus, 1758)	Forest rabbit	Fo; Cb	0.29	0.12	0.18	–	–	5
Cingulata								
Dasypodidae								
*Dasypus novemcinctus* (Linnaeus, 1758)	Armadillo	Fo; Cb; Zoot	0.22	0.06	0.16	Fat (5)	Throat inflammation Cough	3
*Euphractus sexcinctus* (Linnaeus, 1758)	Yellow armadillo	Fo; Cb; Zoot; Art	1.10	0.65	0.45	Fat (5)	Throat inflammation Cough	12
Didelphimorpha								
Didelphidae								
*Didelphis albiventris* Lund, 1840	Brazilian opossum	Fo; Art	0.86	0.43	0.42	–	–	24
Pilosa								
Myrmecophagidae								
*Tamandua tetradactyla* (Linnaeus, 1758)	Collared anteater	Fo; Cb; Zoot; Art	0.63	0.30	0.33	Intestine (7) Skin (7) Nail (7) Fat (7)	Back pain General pains Asthma	6

Method of use for the treatment of diseases: (1) After tanning the leather, you sit on it; (2) melt and drip it on the hurt area; (3) cook it and drink the broth; (4) toast, macerate it, and make a tea; (5) melt and drink with coffee or pure; (6) toast, macerate, and ingest the powder; (7) toast, macerate, and mix with food or drink; (8) eat the meat

Some of the cited species are used for more than one purpose, such as *C. Thous*, which was related to 6 different kinds of use, especially in the local folk medicine, being used to treat 18 pathologies (Table 3), reflecting thus its local importance.

**Fig. 3:**
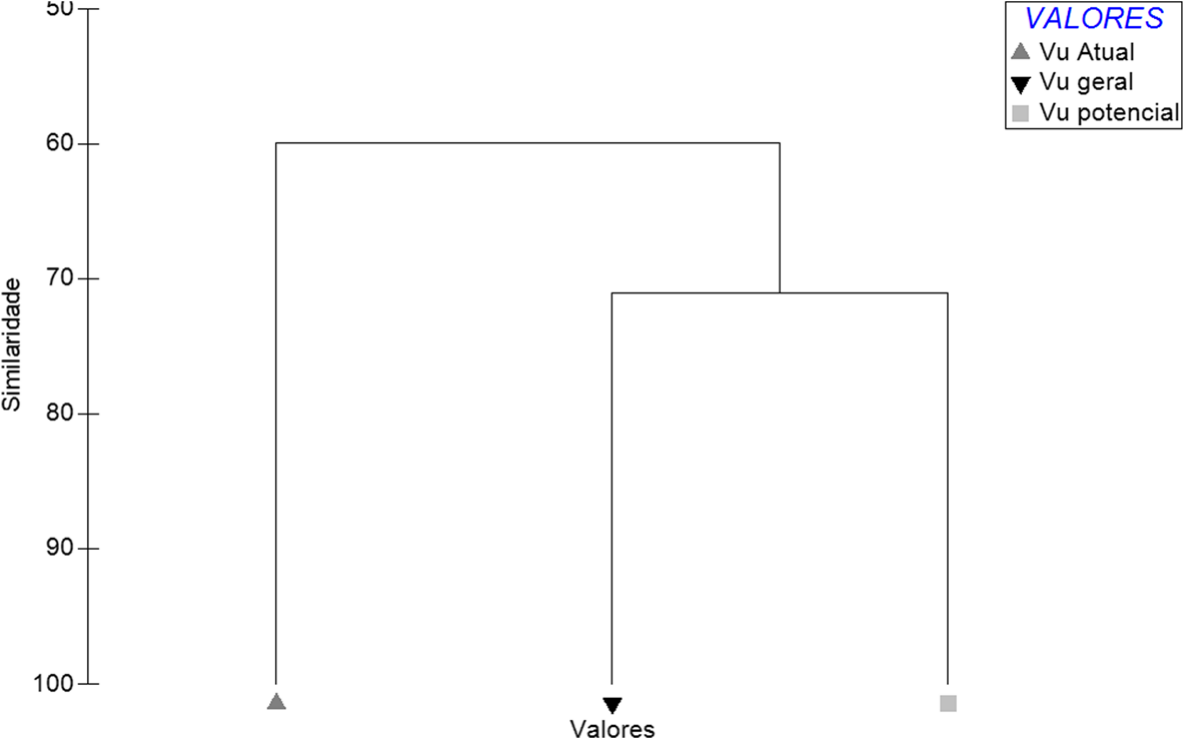
Similarity analysis between the UV_current_, UV_general_, and UV_potential_ of mammal species used in the rural community of Capivara, in the municipality of Solânea (Paraíba State, northeastern Brazil). Gray triangle: UV_current_. Black inverted triangle: UV_general_. Gray square: UV_potential_.

According to the one-way ANOVA, there was a significant difference between the three types of use value calculated for the species used (*F* = 4.048; *P* < 0.02467), especially between the UV_current_ and the UV_general_ (Tukey test *P* < 0.05). This pattern is observed in the similarity analysis, in which the UV_current_ is included in a group different from the other two types of UV (Fig. 3).

**Fig. 4:**
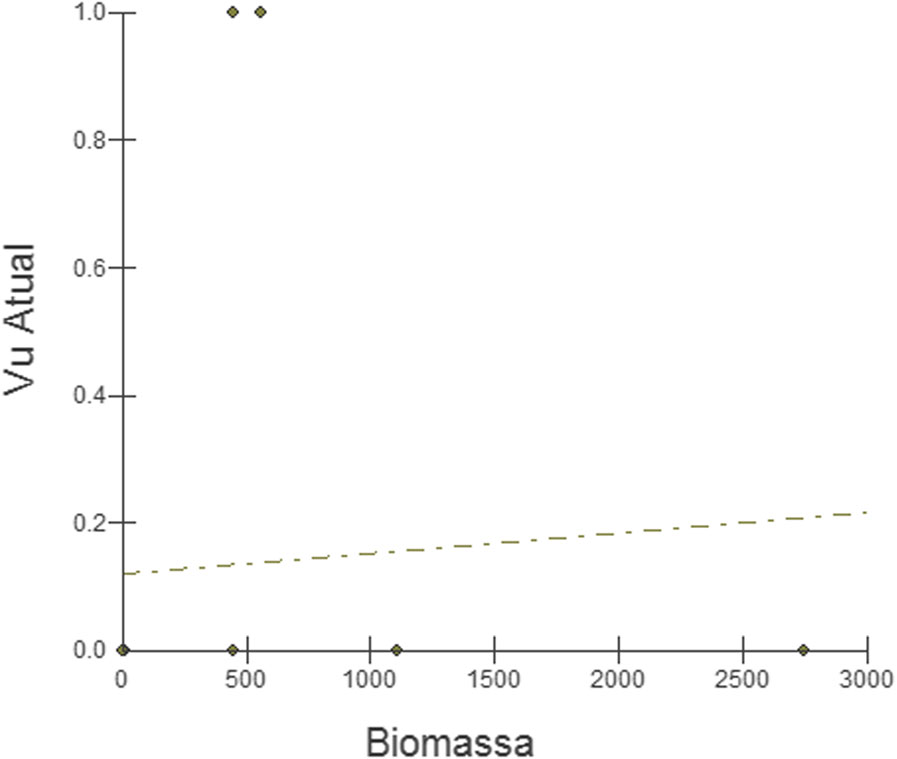
Relationship between the biomass and the UV_current_ of the species used in the rural community of Capivara, in the municipality of Solânea (Paraíba State, northeastern of Brazil)

The results from the regression analysis (Fig. 4) show that there is no significant relationship between the biomass of the consumed wild animals and the UV attributed to them (readjusted = 0.0723; *F* = 0.0558; *p* = 0.39).

## Discussion

The use of mammal species is a practice carried out all over the world [43, 61–66], demonstrating that the use of wild animals is supposed to be culturally disseminated. In the semi-arid region of Brazil, these relationships between humans and nature become even more important, because the environmental factors associated with precarious socioeconomic conditions led these people to develop a very unique sociocultural structure and a strong relationship with the faunistic and floristic resources of their region. This is reported in several studies (e.g., [16, 67–71]), especially during drought periods, when the agriculture and the raising of domestic animals are not feasible due to the lack of resources [16, 42].

Lucena et al. [72] explain that men and women have different patterns of knowledge (similar or not) of the natural resources, depending on the study region. The data obtained in our study indicate that men and women know and use the same species. Concerning the mastery of the categories, men, because they are mostly in contact with nature, work outside the residences, and besides performing cinegetic activities, they are more related to these practices. However, women demonstrate mastery in preparing the animals used in the culinary and those related to folk medicinal treatments. This can be explained by the fact that women are usually responsible for domestic activities and family welfare.

All the species described were attributed to food purpose, obtaining a higher number of citations and higher use values; these animals are important because they are much appreciated in the local cuisine. This trend of preference for some animal species as a food resource has already been observed in some studies in the world [16, 42, 66, 73–77] and also in the studied community, where the hunting is directed to animals of which meat is considered tasty.

Regarding the breeding category, some studies show that this relationship between human populations and animals is common in the semi-arid region of Brazil [12, 24, 78]. In the study area, the species are used as pets due to the affective bond and their beauty (e.g., *Leopardus* sp.) and because they can later be used as food resource (e.g., *Euphractus sexcinctus*) (Fig. 2), reflecting their number of citations. The latter one (food resource) is also reported by Alves et al. [78].

The carnivorous species had a significant number of citations for artisanal purposes, and leather was commonly cited as the material used to make musical items, such as tambourine and zabumba, as well as saddles, motorcycle seats, chair covers, car seats, shoe and boot soles, bags, hats, key rings, and belts, among others. However, none of these ornaments were seen in the visited residences. Thus, the UV_general_ attributed by the interviewees to the species for ornamental purposes, evidences their local importance, even though they are not currently used. Nevertheless, 11 respondents reported executing the use (UV_current_) if the animals are slaughtered.

In addition to the leather, an informant described the use of hoof of *Euphractus sexcinctus* (Linnaeus, 1758) to decorate the residence. Another interviewee reported the use of rennet (stomach) of *K. rupestris* in the preparation of cheese, making the process faster; this trend has already been recorded in other research [78].

The species related to the traditional medicinal use corroborate the list of mammals described by researchers over the years in Brazil [10, 12, 36, 70, 78]. With regard to the magic/religious and veterinary categories, *C. thous* had one citation in each one of them. For the first purpose, the tail is placed on domestic animals (cattle, goats, and sheep) to prevent the approach of bat species. For the traditional veterinary practice, “lard” (animal fat) is melted and then applied to females of domestic animals, such as cows, goats, and sheep, after the delivery process in order to clean the uterus of these animals.

Thus, utility citations relating to the species reflect their general use value, which may or may not be altered when the analysis of the current use value is performed. It should be noted that the low current use value may be related to several factors, such as a reduction in the use and concealment of information, due to the illegal use of wild animals.

Another factor possibly related to this preference, based on the interviews, is justified by two biases: (1) Perceived abundance of these animals, since, when the informants were asked about the abundance of species, *C. thous* and *G. spixii* was described as one of the most found in the region (respectively, 53 and 39 citations referring to high abundance perceived). This data was collected through visual stimulation, using figures (squares) containing different numbers of dots to represent the perceived abundance of the species in the area, seeking to standardize and reduce the subjectivity of the information provided by the interviewees.

(2) Spatial distribution, all the informants (*n* = 108) indicated the different areas where the mentioned species may be found, extending from conserved areas to anthropized areas, since *G. spixii* is found in several environments distinguishable by the informants, such as open field, closed field, quarries, crops of forage cactus, and near the residences, which reflected its high number of citations and higher general use value and current use value. Some studies conducted in traditional communities also reported the identification of ecological zones by the informants (e.g., [79, 80]). Such specialties relating to local ecological knowledge facilitate the collection of specimens (animals), making possible the attribution of utility value to the animals.

Some of the cited species are used for more than one purpose. The multiple uses of a species can be discussed from two different points of view. The former is a conservationist because the use of a species for different purposes means additional uses or hunting and may result in pressure on this species. The more diversified the attributions to a species are, the higher the chance of it being known/used by the population and the higher probability of it being introduced into the culture by replacing an extinct species [18, 24, 78, 81–83].

The second one concerns the optimization of the use of natural resources by the local community, considering that, due to the peculiar socioeconomic situation of the residents, they attempt to make the most of the available resources, since a large part of these by-products (tails, carapace, bones) would be discarded [18, 24, 78, 83, 84]. Thus, the same species can have its by-products used as food, in traditional medicine, to make some items, etc. Mendonça et al. [85], in a study carried out in the semi-arid region of Brazil, reported that it is quite common for local hunters to make the most of the hunted animal parts, resulting in at least two distinct uses for the majority of cinegetic resources, including species considered dangerous to man or to domestic animals.

In the present work, the biomass of the species had no influence on their UV_current_, evidenced in the simple linear regression (the relationship between biomass and the UV_current_). Although according to the Optimal Foraging Theory, local populations prefer to use animals of higher biomass, obtaining a greater quantity of resources per hunting and/or manufacturing time [40, 46, 48], several studies have reported that as the medium- and large-sized species are depleted, typical species with higher availability become the subsequent targets [40, 42, 78, 86–90]. In fact, this is the trend pointed out in ethnobiological studies around the world (e.g., [91–96]). Therefore, small-sized species may be as important for the communities as large-sized species (higher biomass).

Some authors have reported the hunting of small-sized species in other semi-arid areas in Brazil, such as Mendonça et al. [88], who investigated the preference of hunters for species of large, medium, and low body biomass and found that hunters in the semi-arid region have no significant preference for large-sized animals. Therefore, people have developed a peculiar way of dealing with and making the most of available resources, which are often obtained in large quantities, thus making up for the low body weight of species of local importance.

Several cultural and ecological factors are associated with the choice and use of species in the Caatinga [16, 42, 87, 88], which may justify the variations between the different use values attributed to each species (see Table 3). For example, small- (< 1 kg) and medium-sized species (1–5 kg) such as *G. spixii*, *T. apereoides*, *K. rupestris*, *Didelphis albiventris* Lund, 1840 and *Callithrix jacchus* (Linnaeus, 1758) (< 1 kg), *Dasypus novemcinctus* (Linnaeus, 1758), *E. sexcinctus*, and *Sylvilagus brasiliensis* (Linnaeus, 1758) (1–5 kg) had high UV_general_ and UV_current_ (Table 3) and high reproductive and density rates, and except for *Kerodon rupestris* (WIED-NEUWIED, 1820), all these species are generalist as for the use of habitat, are widely distributed, and tolerate anthropogenic disturbances (r-strategists) [9, 97–102].

*K. rupestris* despite being a specialist in using habitats (rocky outcrops and mountainous areas), it has a high reproductive rate and gregarious habit [9, 103]. This species is endemic to the Caatinga, and although it is widely distributed throughout this biome, it is classified as vulnerable on the national list of endangered species, due to the hunting pressure [104].

With regard to *D. novemcinctus* and *E. sexcinctus*, according to Marinho et al. [105], the abundance of these species in areas of Caatinga is very relative and although they are hunted in several areas [12, 16, 35, 42, 70, 78, 88, 106, 107] they are widely distributed in the biome. Other medium-sized species (1–5 kg) such as *Conepatus amazonicus* (Lichtenstein, 1838) and *Tamandua tetradactyla* (Linnaeus, 1758), despite having low population densities and low reproductive rates, are easily found in several areas of the Caatinga [9].

Regarding *C. thous*, it is reported in the literature as a medium-sized animal with the highest number of records in Paraíba, Pernambuco, Ceará, and Alagoas. This species occurs in all habitats, visibly adaptable to anthropized areas, and is found even in green areas of cities [9, 108]. Thus, it is constantly seen, which results in its high perceived abundance, described by the interviewees, and in the consequent association with purposes of use.

With regard to the mesopredator carnivorous (5–10 kg) such as tayras (*Eira barbara*), crab-eating raccoon (*Procyon cancrivorus*), and all Felidae species and top predators such as *Puma concolor* (Linnaeus, 1771) (> 15 kg), some studies have shown that these species already occur in low population densities and have ecological and behavioral peculiarities which make them more vulnerable to anthropogenic pressures [9, 97, 98, 105, 109]. The felines recorded in the present study, except for *Leopardus pardalis* (Linnaeus, 1758), are classified as vulnerable on the national and international lists of endangered species [104, 110]. The state of *P. concolor* is more critical in the Caatinga than it is in the national classification; in this biome, this species is classified as endangered [105, 111].

Another important factor is that although there are many large-sized animals in the Caatinga [9, 105], they are strongly affected by hunting and habitat loss because they are k-specialists and have several trophic requirements, low reproductive rate, long lifetime, and are very susceptible to anthropogenic disturbances [9, 97, 98, 105].

All these factors are aggravated by the increased aridity and increase in areas susceptible to desertification since 20% of the Caatinga is undergoing desertification [106, 112]. This may explain the absence of many other species, evidencing the need for future studies aimed to investigate how wild populations respond to the impacts caused by human activities. It is worth mentioning that, in this ecosystem, most studies on the ecology of species and on factors associated with anthropogenic impacts are still local and incipient [9, 16, 35, 105].

The UV_current_ of the cited species ranged from zero to 1.49 and the UV_potential_ from 0.02 to 1.57. Most of the animals had UVs (general, potential, and current) lower than 1, but eight species had higher UVs, which reflect their local importance to the community. Thus, a high UV_current_ is worrisome in the case of local exploitation; however, a high UV_potential_ may indicate the lack of species in the region [90, 107]. It is worth noting that although species of high potential use values are not effectively used, they can become part of the regular uses as the currently used species are extinct [39, 90–93]. Another relevant consideration is that species with high potential use may be absent in the region, which may explain the recognition and no effective use of potential species [92, 107].

Although we have collected no quantitative data on consumption frequency and number of animals slaughtered, as well as no data on species abundance, frequency, and use of habitat, based on information from local residents and other studies on hunting and use of wild animals in the Caatinga [10, 12, 16, 42, 70, 88], the use of wild mammals is widely disseminated in several areas and is the main cause of population reduction and local extinction of several species [35, 42, 101, 109].

Some studies [90, 107, 113–115] have shown that the application of differentiated UV results in more precise diagnoses regarding the cultural importance of the species used. Thereby, aiming at strategies for the conservation of these animals, further studies are needed to evaluate if the local decrease in the population of some species, from the informants’ point of view, is directly related to their use and/or to other factors, such as population fluctuation and changes in their habitat, among others. These factors may be associated with the capture/use of the species and are fundamental data for the development of conservation strategies.

Although the UV is a data analysis tool widely used in recent ethnobiological studies, a standard method for collecting and analyzing the data on the uses cited by the interviewees has not yet been established. This divergence in obtaining information may provide a margin for crucial errors in the results obtained by the researchers.

The results presented here show the importance of adopting the UV_current_ in the analysis of ethnobiological data since this UV explains the effective use of natural resources and is significantly different from the UV_general_ of the species, based on the Cluster test and one-wayANOVA. No studies had statistically measured this difference, until now, although some authors have pointed out the need to assess the applicability of this index [92, 107, 113–115]. Therefore, the data from this research suggest a new standard for data analysis using the UV_current_ as a strategic tool for the conservation of the most important species. The systematization in data organization works as a support for more reliable comparisons between ethnobiological studies. Thereby, the present ethnozoological study, since its data is quantitatively interpreted, needs to be categorized and delimited to provide reliable results on how these people use natural resources.

## Conclusions

In this context, it is expected that other studies, using this differentiated UV method, systematize the calculation according to each citation described by informants, considering that such information may be situational and modifiable according to the area and time where the research will be developed. However, these studies must be able to point out which species possibly require conservation strategies to minimize negative impacts on biodiversity. Such information becomes increasingly necessary not only for the preservation of the Caatinga but also for other ecosystems in which there is a relationship between humans and natural resources for utilitarian purposes.

The understanding of this technique and its consequent standardization proposed in this article can represent a fundamental reference for future ethnozoological studies and may contribute to the development of management plans and sustainable use of the wild fauna by traditional populations that depend on this resource for survival.

## Data Availability

The authors do not wish to provide data from their studies, as some databases charge fees and there is no proper Brazilian database.
